# The impact of obesity and bariatric surgery on the immune microenvironment of the endometrium

**DOI:** 10.1038/s41366-021-01027-6

**Published:** 2021-12-02

**Authors:** Anie Naqvi, Michelle L. MacKintosh, Abigail E. Derbyshire, Anna-Maria Tsakiroglou, Thomas D. J. Walker, Rhona J. McVey, James Bolton, Martin Fergie, Steven Bagley, Garry Ashton, Philip W. Pemberton, Akheel A. Syed, Basil J. Ammori, Richard Byers, Emma J. Crosbie

**Affiliations:** 1 The University of Manchester Medical School, Oxford Road, Manchester, M13 9PL UK; 2 Division of Gynaecology, St Mary’s Hospital, Manchester University NHS Foundation Trust, Manchester Academic Health Science Centre, Oxford Road, Manchester, M13 9WL UK; 3 Division of Cancer Sciences, School of Medical Sciences, Faculty of Biology, Medicine and Health, University of Manchester, Stopford Building, Oxford Road, Manchester, M13 9PT UK; 4 Gynaecological Oncology Research Group, Division of Cancer Sciences, School of Medical Sciences, Faculty of Biology, Medicine & Health, University of Manchester, St Mary’s Hospital, Oxford Road, Manchester, M13 9WL UK; 5 Department of Pathology, Manchester University NHS Foundation Trust, Manchester Academic Health Science Centre, Oxford Road, Manchester, M13 9WL UK; 6 Division of Informatics, Imaging & Data Sciences, School of Biological Sciences, Faculty of Biology, Medicine and Health, University of Manchester, Stopford Building, Oxford Road, Manchester, M13 9PT UK; 7 CRUK Manchester Institute, The University of Manchester, Alderley Park, Alderley Edge, SK10 4TG UK; 8 Department of Clinical Biochemistry, Manchester University NHS Foundation Trust, Manchester Academic Health Science Centre, Oxford Road, Manchester, M13 9WL UK; 9 Department of Obesity Medicine, Diabetes & Endocrinology, Salford Royal NHS Foundation Trust, Manchester Academic Health Science Centre, Stott Lane, Salford, M6 8HD UK; 10 Division of Diabetes, Endocrinology and Gastroenterology, School of Medical Sciences, Faculty of Biology, Medicine & Health, University of Manchester, Oxford Road, Manchester, M13 9PL UK; 11 Department of Surgery, Salford Royal NHS Foundation Trust, Manchester Academic Health Science Centre, Stott Lane, Salford, M6 8HD UK

**Keywords:** Cancer, Immunology

## Abstract

**Background:**

The incidence of endometrial cancer is rising in parallel with the obesity epidemic. Obesity increases endometrial cancer risk and weight loss is protective, but the underlying mechanisms are incompletely understood. We hypothesise that the immune microenvironment may influence susceptibility to malignant transformation in the endometrium. The aim of this study was to measure the impact of obesity and weight loss on the immunological landscape of the endometrium.

**Methods:**

We conducted a prospective cohort study of women with class III obesity (body mass index, BMI ≥ 40 kg/m^2^) undergoing bariatric surgery or medically-supervised low-calorie diet. We collected blood and endometrial samples at baseline, and two and 12 months after weight loss intervention. Serum was analysed for inflammatory markers CRP, IL-6 and TNF-α. Multiplex immunofluorescence was used to simultaneously identify cells positive for immune markers CD68, CD56, CD3, CD8, FOXP3 and PD-1 in formalin-fixed paraffin-embedded endometrial tissue sections. Kruskal–Wallis tests were used to determine whether changes in inflammatory and immune biomarkers were associated with weight loss.

**Results:**

Forty-three women with matched serum and tissue samples at all three time points were included in the analysis. Their median age and BMI were 44 years and 52 kg/m^2^, respectively. Weight loss at 12 months was greater in women who received bariatric surgery (*n* = 37, median 63.3 kg) than low-calorie diet (*n* = 6, median 12.8 kg). There were significant reductions in serum CRP (*p* = 3.62 × 10^−6^, *r* = 0.570) and IL-6 (*p* = 0.0003, *r* = 0.459), but not TNF-α levels, with weight loss. Tissue immune cell densities were unchanged except for CD8+ cells, which increased significantly with weight loss (*p* = 0.0097, *r* = −0.323). Tissue CD3+ cell density correlated negatively with systemic IL-6 levels (*p* = 0.0376; *r* = −0.318).

**Conclusion:**

Weight loss is associated with reduced systemic inflammation and a recruitment of protective immune cell types to the endometrium, supporting the concept that immune surveillance may play a role in endometrial cancer prevention.

## Introduction

Endometrial cancer is the most common gynaecological malignancy in the United Kingdom and its incidence is rising [1]. Obesity is a major risk factor for the disease, with every 5 kg/m^2^ increase in body mass index (BMI) conferring a 1.6-fold higher risk [2]. Women with a BMI > 40 kg/m^2^ are particularly vulnerable, with lifetime risks as high as 10–15% [3]. With 58% of the world population estimated to be overweight or obese by the year 2030 [4], the burden of endometrial cancer is set to continue [5]. Whilst most are detected early and cured by hysterectomy, women with high risk or late stage endometrial cancer have poor outcomes [6]. An improved understanding of the biological mechanisms underpinning obesity-associated endometrial cancer could lead to new targets for prevention and treatment [7]. Obesity is thought to drive endometrial carcinogenesis through adipose-derived oestrogen, which, when unopposed by progesterone in postmenopausal and premenopausal anovulatory women, promotes endometrial proliferation and tumourigenesis [8]. Other contributors include metabolic dysfunction, insulin resistance and hyperinsulinaemia, factors that further stimulate endometrial growth through direct and indirect pathways [9]. Obesity is a chronic pro-inflammatory state, with increased circulating levels of the inflammatory markers C-reactive protein (CRP), interleukin-6 (IL-6) and tumour necrosis factor-alpha (TNF-α) [10]. These inflammatory markers may mediate alterations in the immune microenvironment of the endometrium that support neoplastic transformation.

Weight loss achieved and sustained through bariatric surgery or dietary intervention has been shown to reduce endometrial cancer risk [11–14]. In a prospective study of women undergoing gastric bypass or sleeve gastrectomy-induced weight loss, we found occult endometrial cancer or its precursor lesion, atypical hyperplasia, in 14% of those sampled at baseline [15]. Spontaneous resolution of atypical hyperplasia was observed 2 months following bariatric surgery in 3/6 women and a further 2/6 were successfully managed with intrauterine progestin. In those with histologically normal endometrium, we observed a weight loss-associated down-regulation of pro-proliferative signalling pathways, including reductions in proliferation marker Ki-67. These findings support the hypothesis that weight loss instigates changes in the endometrium that influence its susceptibility to malignant transformation. We propose that the immune microenvironment is a dynamic regulator of endometrial health that is perturbed in obesity and restored with weight loss. The aim of this study was to measure the impact of obesity and weight loss on the immunological landscape of the endometrium using matched serum and endometrial tissue samples from our cohort of women undergoing intentional weight loss.

## Methods

### Study participants

This was a prospective cohort study of women with morbid obesity (BMI ≥ 40 kg/m^2^) undergoing weight loss management at Salford Royal NHS Foundation Trust [15]. Approval was obtained from the North West Research Ethics Committee (12/NW/0050) and the study was prospectively registered (ISRCTN17241389). All participants gave written, informed consent. Height was measured using a stadiometer, weight was measured using electronic scales after removal of bulky clothing, and BMI derived (kg/m^2^). Blood and endometrial tissue were collected at baseline, and two and 12 months following medically-supervised low-calorie diet or bariatric surgery, either gastric bypass, sleeve gastrectomy or gastric banding. Postmenopausal status was defined as last menstrual bleed >12 months previously. Premenopausal participants were sampled in the late proliferative phase, except for baseline biopsies of women undergoing bariatric surgery, which were obtained under general anaesthetic. Endometrial sampling was by Pipelle^©^ (Carefusion, UK) or MedGyn Endosampler^©^ (MedGyn, Illinois, USA). Tissue was fixed in 10% neutral buffered formalin prior to embedding in paraffin wax. Histopathological interpretation of haematoxylin and eosin-stained sections was by specialist gynaecological pathologists (RJM and JB) according to the WHO classification system [16, 17]. Serum IL‐6 and TNF-α were measured using a DuoSet ELISA development kit (R&D Systems, Abingdon, UK) and high sensitivity CRP (hsCRP) was measured by an in‐house antibody sandwich ELISA technique with antihuman CRP primary antibodies from Abcam (Cambridge, UK).

### Immunohistochemistry

Four-micron sections were cut from formalin-fixed paraffin-embedded tissue samples and multiplex immunofluorescent staining was performed using a Ventana Autostaining Robot (Ventana Medical Systems, Arizona, USA), with automation of deparaffinisation, antigen retrieval and incubation but manual application of reagents. Antibodies (Table S1) were applied sequentially in the following order, with fluorescent disclosure of each antibody with a different fluorophore, and antibody denaturation between each primary antibody application: CD8, CD68, CD3, FOXP3, CD56, PD-1. Following multiplex staining, all sections were cover-slipped with the Prolong aqueous mounting agent (Thermo Fisher, Massachusetts, USA) that also contained DAPI for counter-staining. The detailed multiplex staining protocol is given in Supplementary Methods.

### Tissue imaging and analysis

Stained sections were captured by slide scanning and image acquisition using the Vectra 3.05 multispectral imager (PerkinElmer, Massachusetts, USA). Each section was first scanned at a magnification of 10x, producing a low-resolution overview of the tissue. Up to 10 regions of interest were randomly sampled from the low-resolution overview image, using Phenochart whole slide viewer (PerkinElmer, Massachusetts, USA). These regions were then scanned at a higher magnification of ×20 (0.496 μm/pixel). Each scanned region consisted of nine multispectral images (MSI) that were automatically stitched together.

**Fig. 1:**
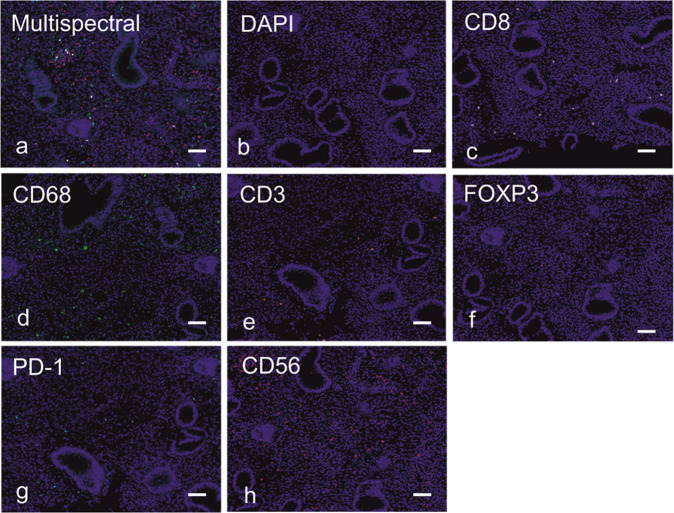
Multiplex immunofluorescence and automated image analysis to measure tumour infiltrating immune cells. Each of (**a**–**h**) correspond to the same tissue region, demonstrating the raw unmixed multispectral image containing all immunofluorescent stains (**a**) and resultant separately resolved cellular populations after spectral unmixing in (**c**–**h**), specifically in (**c**) CD8+ cells, (**d**) CD68+ cells, (**e**) CD3+ cells, (**f**) FOXP3+ cells, (**g**) PD-1+ cells, and (**h**) CD56 + cells; DAPI nuclear counterstain shown in (**b**) was used for nuclear segmentation and resultant cellular segmentation; magnification × 200 in all panels and scale bar 100 microns. All markers are displayed using pseudo-colours.

MSI were unmixed and analysed in three stages: spectral unmixing, cell segmentation and cell phenotyping. Linear spectral unmixing was carried out using InForm 2.4 software (PerkinElmer, Massachusetts, USA). For unmixing, a spectral library was built comprising individual fluorophore spectra. Each spectrum was acquired from slides that were single stained for each antibody, using the same experimental parameters as the multiplex experiment. A slide stained with DAPI only was used to extract the DAPI spectrum. A slide that underwent all steps in the multiplex experiment without application of antibodies or fluorophores was used to extract the spectrum of tissue auto-fluorescence. After spectral unmixing, six resultant images were generated representative of the staining intensities of each fluorophore (Fig. 1) in which the intensity at each pixel is proportional to the quantity of fluorophore and its corresponding epitope, at that pixel.

Cell segmentation was conducted using Inform software. A two-step process was carried out using only the DAPI channel. First, nuclear components were detected using nuclear stain intensity. Then the cytoplasm around each nucleus was simulated by cell expansion of 2 μm and measurements generated for marker intensity in different compartments (mean, minimum, maximum and standard deviation of intensity in cytoplasm or nucleus).

Cell scoring was carried out using an in-house software developed in the imaging department of the University of Manchester [18]. The software automatically determines if a cell is positive or negative for each stain using a pre-determined threshold cut-off. Positivity for each cell type was determined by the intensity of each marker in the primary cell compartment where it was usually expressed. In our study, markers were cytoplasmic or membranous. Before cell scoring, the intensity of each marker was re-scaled onto a grey-scale colour map, where the brightest and darkest values corresponded to the 99% and 1% percentiles of the marker’s pixel intensities in the full dataset. Having a consistent colour-map per marker ensured that the same intensity value was represented with equal brightness in all images.

Guided by a pathologist (RB), a single threshold for each marker was selected as a cut-off to determine positivity across the entire dataset. The threshold was identified by its ability to separate positive from negative cells in a set of 20 regions of interest from different patients. The resultant number of positive cells per unit area was measured in each of the patient samples for each of the antigens assayed.

### Statistical analysis

All statistical analyses were conducted in R 3.5.1 × 64/RStudio 1.0.143 × 64. To determine suitability of data for parametric testing, QQ normality plots were called for both response variables and response variable linear model residuals vs. standardised residuals for the six tissue immune markers (CD8, CD68, CD3, FOXP3, CD56, PD-1), three serum inflammatory markers (hsCRP, IL‐6, TNF-α) and clinical parameters (BMI and weight). Only BMI and weight held distributions approaching normality. Homogeneity of variance was probed by Barlett’s Test followed by Levene’s Test. Fligner-Killeen tests were used for variables determined to have departures from normality. All variables except for FOXP3, CRP, TNF-α and BMI demonstrated heteroscedastic variances.

Normality and variance violations prohibited parametric analysis of data, but only 7% of observations were suitable for BoxCox transformation into normal distributions. Kruskal–Wallis tests were therefore used to determine if there were any significant changes in immune, inflammatory and clinical biomarkers between baseline and 2 months, and between baseline and 12 months. Dunn’s multiple comparisons post hoc was implemented to determine groups with significant differences with a Sidak or Benjamini Hochberg multiple comparisons adjustment.

Time series correlation modelling explored relationships between tissue and serum biomarkers. Nonlinear mixed effect (nlme) modelling was trialled to accommodate distribution differences, unequal class sizes, autocorrelation, and heteroscedastic variances; however, not all markers successfully converged using this method. Repeated measures correlation modelling was therefore used since it protects against class imbalance, correlated error rates and heteroscedasticity. To alleviate requirements for normally distributed data we bootstrapped variable-specific distributions during model generation.

## Results

### Study participants

From a total cohort of 80 participants, 43 had matched serum and tissue samples at baseline, and 2 months and 12 months after weight loss intervention and were included in this study. Their median age and BMI at baseline were 44 years (range 24–60 years) and 52 kg/m^2^ (range 38–69 kg/m^2^), respectively. Most (37/43, 86.0%) were premenopausal. Thirty-seven women underwent bariatric surgery, either gastric bypass (26/37, 70.3%), sleeve gastrectomy (11/37, 29.7%) or gastric banding (1/37, 2.7%), and 6/43 (14%) underwent medically-supervised low-calorie diet. At baseline, six women had occult atypical endometrial hyperplasia, five of which resolved with weight loss and/or intrauterine progestin, as previously described [15]. Median weight loss was greater for women who underwent bariatric surgery compared to those who followed a low-calorie diet, both at 2 months [−15.1 kg (MAD 4.59, *p* = 0.0201) vs. −1.7 kg (MAD 2.48 kg, *p* = 0.936) respectively] and 12 months [−63.3 kg (MAD 17.64, *p* < 0.0001) vs. −13.0 kg (MAD 11.1, *p* = 0.0679) respectively]. Median BMI was 53 kg/m^2^ and 47.1 kg/m^2^ at baseline, 45 kg/m^2^ (*p* = 0.0002) and 46.7 kg/m^2^ (*p* = 0.936) at 2 months and 35 kg/m^2^ (*p* < 0.0001) and 43.6 kg/m^2^ (*p* = 0.201) at 12 months in women undergoing bariatric surgery and low-calorie diet, respectively.

**Fig. 2:**
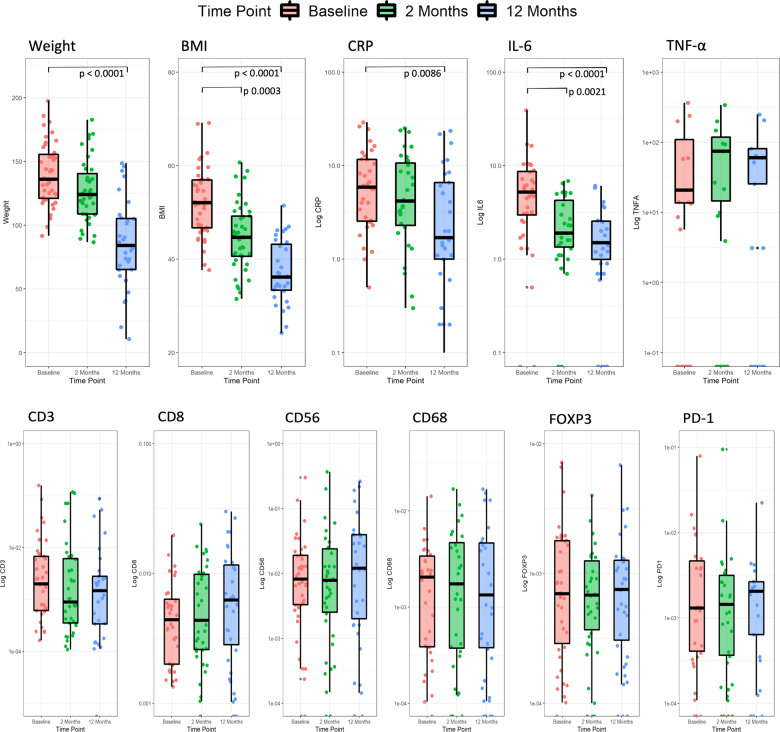
Change in clinical, inflammatory and immune biomarkers with time. Box and jitter plots for biomarkers across time points, including weight, BMI, systemic inflammatory and immune cell marker densities. *Y* axes are log scale. Significant *p* values are provided at bracketed comparators where relevant (Kruskal–Wallis tests with Dunn–Šidák corrections). The α for all tests was 0.05. Significant reductions were observed for weight between baseline: 12 months (*p* < 0.0001); BMI between baseline: 2 months (*p* = 0.0003) and baseline: 12 months (*p* < 0.0001); CRP between baseline: 12 months (*p* = 0.0086); and for IL-6 baseline: 2 months (*p* = 0.0021) and baseline: 12 months (*p* < 0.0001).

**Fig. 3:**
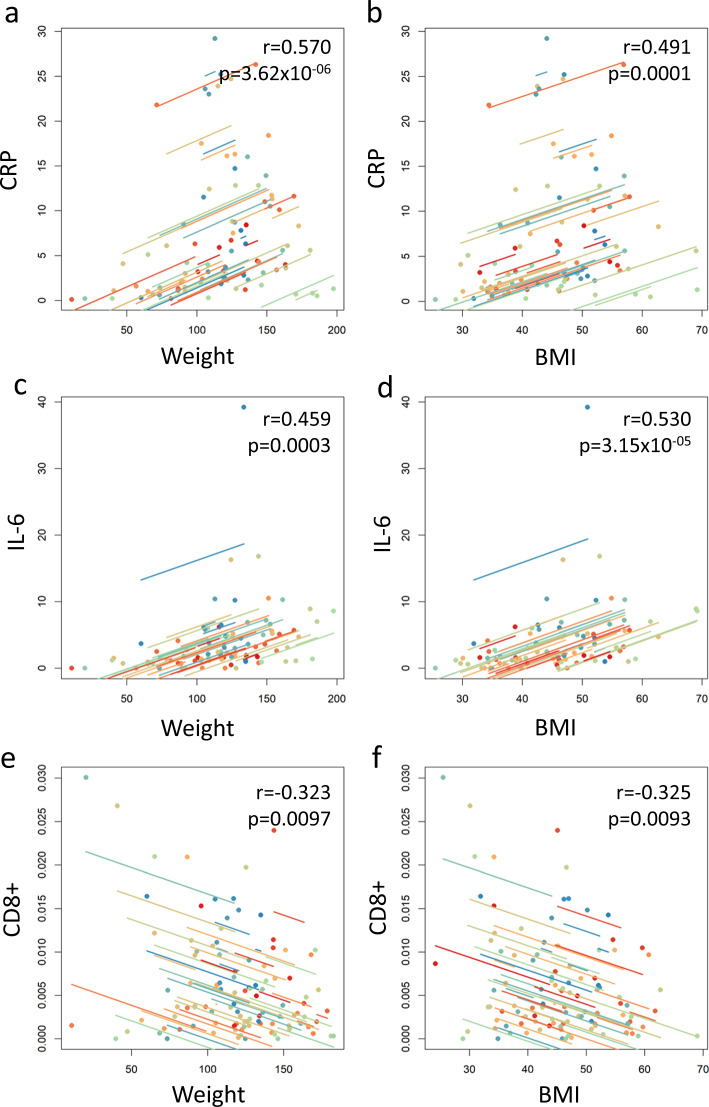
Correlations between weight, BMI, inflammatory biomarkers and immune infiltrates. Repeated measures correlation plots are shown. Significant positive correlations were observed between reduced systemic inflammatory marker CRP and reduced weight (**a**) or BMI (**b**) (*r* = 0.57, *p* = 3.62 × 10^−6^ and *r* = 0.491, *p* = 0.0001, respectively). Significant positive correlations were observed between reduced systemic inflammatory marker IL-6 and reduced weight **c**) or BMI (**d**) (*r* = 0.459, *p* = 0.0003 and *r* = 0.530, *p* = 3.15 × 10^−5^, respectively). Significant negative correlations were observed between increased CD8+ immune cells and reduced weight (**e**) or BMI (**f**) (*r* = −0.323, *p* = 0.0097 and *r* = −0.325, *p* = 0.0093, respectively). Repeated measures correlation distributions were bootstrapped from data structures to accommodate potential unequal variance and distributions within the time dependent analysis. The α for all tests was 0.05.

Systemic inflammatory markers CRP, IL-6 and TNF-α were mildly elevated at baseline (Table 1). Median CRP was significantly lower at 12 months (*p* = 0.0086) compared to baseline (Fig. 2). CRP correlated significantly with weight loss (*p* = 3.62 × 10^−6^, *r* = 0.570) and BMI reduction (*p* = 0.00014, *r* = 0.491) (Fig. 3). Median IL-6 showed significant reductions at 2 months (*p* = 0.0021) and 12 months (*p* < 0.00001) compared to baseline (Fig. 2), which correlated with weight loss (*p* = 0.00032, *r* = 0.459) and BMI reduction (*p* = 3.15 × 10^−5^, *r* = 0.53) (Fig. 3). TNF-α levels did not change significantly over time, with weight loss or with BMI reduction.

**Table 1:** Systemic inflammatory markers in matched serum samples.

Serum biomarker	Baseline (median)	2 months (median)	12 months (median)	Baseline – 2 months	Baseline – 12 months
CRP (μg/ml)	5.9	4.2	1.7	*p* = 0.5572	***p*** < **0.0086**^**a**^
IL-6 (pg/ml)	4.8	1.7	1.2	***p*** = **0.0021**^**a**^	***p*** < **0.0001**^**a**^
TNF-α (pg/ml)	29.1	31.4	25.2	*p* = 0.7307	*p* = 0.8609

^a^Denotes statistically significant result.

Bold values indicates statistically significant *p* < 0.05 values.

**Fig. 4:**
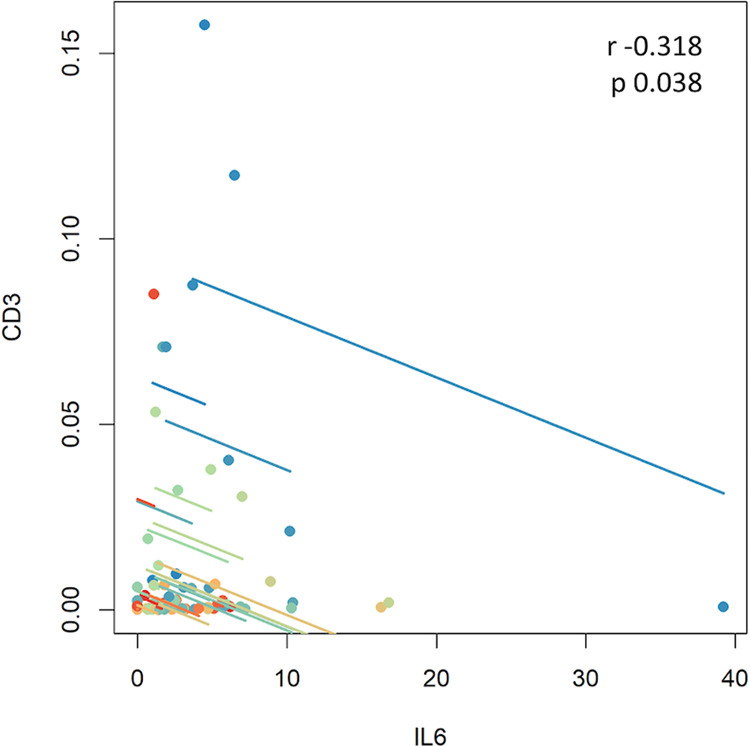
Inverse correlation of CD3+ immune cell infiltrate density with IL-6 across time points. A repeated measures correlation plot of Log CD3+ immune cell density vs. IL-6 intensity. A significant inverse correlation was observed (*p* = 0.038, *r* = −0.318). Repeated measures correlation distributions were bootstrapped from data structure to accommodate potential unequal variance and distributions within the time dependent analysis. The α for all tests was 0.05.

The density of endometrial CD56, CD68, CD3, FOXP3, CD56 and PD-1-positive immune cells at baseline, 2 months and 12 months after weight loss intervention is shown in Table 2 and Fig. 2. The density of CD8 + immune cells was higher at 2 months and 12 months compared to baseline (Table 2) and inversely correlated with weight (*p* = 0.0097, *r* = −0.323) and BMI (*p* = 0.0093, *r* = −0.325) (Fig. 3). The density of CD68+ , FOXP3+ , PD-1+ and CD56+ immune cells showed no significant change with time (Table 2, Fig. 2) nor correlation with weight loss or BMI. The density of CD3+ immune cells was inversely correlated with systemic IL-6 levels (*p* = 0.037; *r* = −0.318) (Table 3, Fig. 4). No significant associations were seen between the other markers (Table 3). Positive associations between CD3 and CD68-positive immune cell infiltrates and systemic CRP levels did not reach statistical significance; neither did the positive association between CD68+ immune cells and systemic TNF-α levels.

**Table 2:** Endometrial immune cell infiltrates in matched tissue samples.

Tissue immune cell biomarker	Baseline Median % positive cells	2 months Median % positive cells	12 months Median % positive cells	Baseline – 2 months	Baseline – 12 months
CD56	0.0071	0.0069	0.0116	*p* = 0.7978	*p* = 0.7737
CD68	0.0019	0.0012	0.0008	*p* = 0.7490	*p* = 0.6634
CD3	0.0019	0.0009	0.0013	*p* = 0.3485	*p* = 0.1717
CD8	0.0037	0.0038	0.0056	*p* = 0.3074	*p* = 0.3016
FOXP3	0.00069	0.00069	0.00066	*p* = 0.6672	*p* = 0.6445
PD-1	0.0011	0.0008	0.0012	*p* = 0.5822	*p* = 0.4360
Median total number of cells counted/sample	14,675	7966	9749	Not applicable

**Table 3:** Correlation of systemic inflammatory markers with endometrial immune cell infiltrates.

	CRP	IL-6	TNF-α
CD8+	0.7720	0.1658	0.3747
CD68+	0.1852	0.4618	0.1473
CD3+	0.2539	**0.0375**^**a**^	0.48457
FOXP3+	0.8527	0.7855	0.7209
CD56+	0.6401	0.9321	0.4723
PD-1+	0.5295	0.8424	0.8989

Table shows *p* values.

^a^Denotes statistically significant result.

## Discussion

Obesity increases endometrial cancer risk and weight loss is protective, but the underlying mechanisms are incompletely understood. Here, multiplex fluorescence immunohistochemistry was used to quantify and phenotype immune cells in the endometrium before and after weight loss in women with class III obesity. There was a significant rise in CD8+ immune cell density at 2 and 12 months that correlated with weight loss. We also found significant reductions in systemic inflammatory markers CRP and IL-6 with weight loss, the latter inversely correlated with CD3+ immune cell density in the endometrium. Taken together, these findings indicate that the immunological landscape of the endometrium may be affected by obesity and weight loss, and by extension, that the immune microenvironment may influence susceptibility to neoplastic change.

Previous work has shown a strong positive correlation between serum levels of pro-inflammatory cytokines and adipokines and endometrial cancer risk [19]. In a prospective study of 107 women sampled before and after bariatric surgery, Linkov et al. (2017) reported near normalisation of serum CRP and IL-6 levels, amongst other pro-inflammatory biomarkers, and concluded that their change may reflect the protective effect of weight loss on endometrial cancer risk [20]. We found one previous study investigating the impact of obesity and weight loss on the immunological landscape of the endometrium [21]. In paired endometrial samples before and 12 months after bariatric surgery-induced weight loss, the authors found no significant difference in CD3+ or CD20+ immune cell density on tissue microarrays for a subset of their overall study population. They did not measure changes in macrophage (CD68), natural killer cell (CD56), cytotoxic T cell (CD8), regulatory T cell (FOXP3) or programmed cell death protein 1 (PD-1) expressing immune cell populations and were therefore not able to make firm conclusions as to the impact of obesity and weight loss on the endometrial immune microenvironment.

Here, we show convincing evidence of a change in the immunological landscape of morphologically normal endometrium in women with class III obesity who undergo significant weight loss. The reduction in systemic inflammation that accompanies weight loss is associated with a recruitment of protective immune cell types to the endometrium, in particular CD8+ cytotoxic T cells. This is consistent with previous work comparing circulating immune cell populations in obese and lean study participants that found higher CD8+ T cell counts in lean individuals [22]. CD8+ T-cells are essential mediators of cancer immune surveillance, keeping tissues healthy through the recognition and selective elimination of cells expressing cancer-specific antigens [23]. An influx of these highly specialised anti-cancer immune cells to the endometrium with weight loss suggests they may have a role in restoring endometrial health and protecting against endometrial cancer. It is already well established that high volume tumour infiltrating CD8+ lymphocytes portend good survival outcomes from colorectal [24], melanoma [25] and many other tumour types [26], including endometrial cancer [27, 28]. Extrapolating these findings to a role for CD8+ T cells in maintaining endometrial health, restoring health through risk-reducing interventions e.g. weight loss, and preventing cancer is an appealing concept worthy of further investigation. It is interesting that systemic IL-6 levels were inversely correlated with endometrial CD3+ T cells since the cancer-promoting properties of IL-6 are well known. It has been shown to stimulate growth, DNA methylation and metastasis and to destabilise the immune microenvironment [29]. Its reduction with weight loss may therefore encourage the recruitment of CD3+ and other protective immune cells to the endometrium. A favourable immunological landscape may enable the natural clearance of latent endometrial precursor and pre-cancer lesions, protecting endometrial health and preventing endometrial cancer [30].

This is the first study, to our knowledge, that compares immune biomarkers in serum and endometrial tissue before and after weight loss in women with class III obesity. Serial measurements across three time points add to the strength of the findings and distinguish short- and long-term effects of weight loss. It is interesting that changes to blood and tissue immune biomarkers were observed as early as 2 months after weight loss intervention, before the majority of excess body weight had been shed. Changes to the immune microenvironment coincided with the natural clearance of atypical hyperplasia in 3/6 women with occult endometrial abnormalities at baseline, indicating their potential role in the restoration of endometrial health. Indeed, resolution of 5/6 endometrial abnormalities occurred by 6 months post-bariatric surgery, before women had achieved a healthy body weight. Immune cell infiltrates vary with menstrual cycle phase and menopausal status [31], and this was controlled for where possible. Multiplex immunofluorescence labelling enabled simultaneous quantification and phenotyping of immune cell markers using a single tissue section. This is advantageous for analysis of small biopsy samples and enables an appreciation of the interplay between different immune cell types within different tissue compartments.

A limitation of our work is the relatively small sample set; however, it derives from a larger cohort of 80 participants and highlights the challenge of longitudinal studies involving sequential invasive sampling. We were unable to control for potential confounding factors, particularly age and menopausal status, and the numbers were too small to allow subgroup analysis by weight loss intervention. Although interesting, our findings are correlative and their significance is uncertain. We were limited by the number of fluorophores we could multiplex in one experiment, precluding the detailed phenotyping of immune cell sub-populations, for example M1 and M2 macrophages. Endometrial recruitment of protective M2 macrophages with weight loss may have been masked by the simultaneous loss of pro-inflammatory M1 macrophages, for instance, and our study design did not allow these cellular sub-populations to be differentiated from each other. Other immune cells may also be important in regulating endometrial health and more research is needed.

If validated in larger studies, the insights provided here could have important clinical implications. Immune biomarkers could enable individualised endometrial cancer risk prediction and also measure the success of risk-reducing interventions. Research could be directed towards encouraging recruitment of protective immune cells to the endometrium to boost the natural clearance of precursor and precancerous lesions. We urgently need innovative solutions to avert the impending surge of endometrial cancer diagnoses predicted by the escalating global obesity problem.

## Supplementary information

Supplemental Material
