# Supplementary material for: The impact of obesity and bariatric surgery on the immune microenvironment of the endometrium
Source: Int J Obes (Lond). 2021 Dec 2;46(3):605–12. doi: 10.1038/s41366-021-01027-6 (PMC8872994; doi:10.1038/s41366-021-01027-6)
Supplement: Supplementary file 1 — Supplemental Material [file 41366_2021_1027_MOESM1_ESM.docx]

**Table S1**

| **Antibody** | **Species** | **Reactivity** | **Clonality** | **Manufacturer** | **Reference number** | **Marker type** |
| --- | --- | --- | --- | --- | --- | --- |
| CD3 | Mouse | Human | Monoclonal | Dako, California, USA | M7254 | T cell marker, co-receptor |
| CD8 | Mouse | Human | Monoclonal | Dako, California, USA | M7103 | T cell marker |
| CD68 | Mouse | Mouse, rat, rabbit, human | Monoclonal | Abcam, Cambridge, UK | ab955 | Macrophage marker |
| FOXP3 | Mouse | Mouse, human, monkey | Monoclonal | Abcam, Cambridge, UK | ab20034 | Marker for Tregs |
| PD-1 | Mouse | Human | Monoclonal | Abcam, Cambridge, UK | ab52587 | T cell marker, T cell suppression |
| CD56 | Rabbit | Human | Monoclonal | Cell Marque, California, USA | 156R-94 | Marker for NK cells |

**Supplementary Methods**

| Step 1 | Tissue is deparaffinised at 69°C for three eight-minute cycles. |
| --- | --- |
| Step 2 | Slide is heated to 95°C for four minutes. Heat-induced antigen retrieval (HIER) is carried out using TRIS–ethylenediamine tetracetic acid (EDTA)–boric acid pH 8 buffer, Cell Conditioner 1(CC1). Slide is then incubated for 64 minutes. |
| Step 3 | Enzyme DISCOVERY Inhibitor (Roche Tissue Diagnostics) is added to the slide and incubated for eight minutes. |
| Step 4 | 110µl of anti-CD8 at the optimised concentration is manually applied to the slide and incubated for 32 minutes at 36°C. |
| Step 5 | 110µl of anti-mouse anti-rabbit secondary HRP from Opal kit is manually applied to the slide and incubated for eight minutes at 37°C. |
| Step 6 | 110 µl of Opal540 fluorophore is manually applied and incubated for 16 minutes.  Antibody denaturation CC2-1 at 90°C for eight minutes.  110µl of anti-CD68 at the optimised concentration is manually applied to the slide and incubated for 32 minutes at 37°C. |
| Step 7 | 110µl of anti-mouse anti-rabbit secondary HRP from Opal kit is manually applied to the slide and incubated for eight minutes at 37°C. |
| Step 8 | 110 µl of Opal520 fluorophore is manually applied and incubated for 16 minutes. |
| Step 9 | Antibody denaturation CC2-2 at 95°C for eight minutes. |
| Step 10 | 110µl of anti-CD3 at the optimised concentration is manually applied to the slide and incubated for 16 minutes at 36°C. |
| Step 11 | 110µl of anti-mouse anti-rabbit secondary HRP from Opal kit is manually applied to the slide and incubated for eight minutes at 37°C. |
| Step 12 | 110 µl of Opal620 fluorophore is manually applied and incubated for 16 minutes. |
| Step 13 | Antibody denaturation CC2-3 at 90°C for eight minutes. |
| Step 14 | 110µl of anti-FOXP3 at the optimised concentration is manually applied to the slide and incubated for 60 minutes at 36°C. |
| Step 15 | 110µl of anti-mouse anti-rabbit secondary HRP from Opal kit is manually applied to the slide and incubated for eight minutes at 37°C. |
| Step 16 | 110 µl of Opal570 fluorophore is manually applied and incubated for 16 minutes. |
| Step 17 | Antibody denaturation CC2-4 at 90°C for eight minutes. |
| Step 18 | 110µl of anti-PD-1 at the optimised concentration is manually applied to the slide and incubated for 60 minutes at 36°C. |
| Step 19 | 110µl of anti-mouse anti-rabbit secondary HRP from Opal kit is manually applied to the slide and incubated for eight minutes at 37°C. |
| Step 20 | 110 µl of Opal650 fluorophore is manually applied and incubated for 16 minutes. |
| Step 21 | Antibody denaturation CC2-5 at 90°C for eight minutes. |
| Step 22 | 110µl of anti-CD56 at the optimised concentration is manually applied to the slide and incubated for 32 minutes at 36°C. |
| Step 23 | 110µl of anti-mouse anti-rabbit secondary HRP from Opal kit is manually applied to the slide and incubated for eight minutes at 37°C. |
| Step 24 | 110 µl of Opal690 fluorophore is manually applied and incubated for 16 minutes |
| Step 25 | Slide is then removed from the Ventana and washed in EZ prep solution for 15 minutes, refreshing the solution every five minutes. Slide is then washed in distilled water for a further five minutes. |
| Step 26 | One drop of DAPI is applied per slide and incubated for five minutes. |
| Step 27 | Slide is rinsed in distilled water for two minutes. |
| Step 28 | Slides are coverslipped using Prolong Gold Anti-Fade Reagent. |
